# The Morphology, Mechanical and Dynamic Properties, Fire Hazard and Toxicity of Chloroprene and Butadiene Rubber Composites Cross-Linked with Zinc

**DOI:** 10.3390/ma16031240

**Published:** 2023-01-31

**Authors:** Aleksandra Smejda-Krzewicka, Przemysław Rybiński, Dariusz Bradło, Witold Żukowski

**Affiliations:** 1 Institute of Polymer and Dye Technology, Lodz University of Technology, Stefanowskiego 16, 90-537 Lodz, Poland; 2 Institute of Chemistry, The Jan Kochanowski University, Żeromskiego 5, 25-369 Kielce, Poland; 3 Department of General and Inorganic Chemistry, Cracow University of Technology, Warszawska 24, 31-155 Cracow, Poland

**Keywords:** elastomer composites, cross-linking, vulcanization, zinc, morphology, tensile strength, Payne effect, fire resistance, fire hazard, toxicity, oxygen index, cone calorimetry

## Abstract

This paper presents the influence of zinc on the cross-linking process, mechanical and dynamic properties, morphologies and balance of thermal degradation of blends containing chloroprene rubber (CR) and butadiene rubber (BR). The novel aspect of this research is a comprehensive approach presenting a new curing agent for the CR/BR blends to increase their cross-linking density and final properties, including non-flammability and low fire hazard. This is due to the need to find an alternative to zinc oxide, which is the standard curing agent for chloroprene rubber. The regulations of the European Union enforce a significant limitation on the use of this compound in elastomer technology, due to its harmful effect on aquatic organisms. In this paper, the CR/BR composites were cured with zinc and filled with natural silica fillers (sillitin or chalcedonite) or synthetic silica filler (aerosil). The investigation focused on the morphology characterization of the obtained compounds, their cross-linking degree, swelling, mechanical and dynamic properties, fire hazard and toxicity. The structure of cured CR/BR blends was characterized via scanning electron microscopy (SEM). The fire resistance studies were performed using cone calorimetry or oxygen index methods, whereas toxicity tests were performed with the use of the FB-FTIR (fluidized bed reactor coupled with FTIR analyzer) method. The results showed that obtained CR/BR products were characterized by satisfactory final properties. The properties determined by the oxygen index and cone calorimetry methods, including the behaviors of the tested CR/BR vulcanizates in fire conditions, showed that the produced compounds were characterized by a low fire hazard and can be classified as non-combustible rubber products. However, the toxicity of the decomposition products, determined at 450, 550 and 750 °C, was very high.

## 1. Introduction

The idea of the cross-linking (vulcanization) of elastomers (rubbers) is to produce intermolecular cross-links between linear or branched rubber macromolecules leading to an effective three-dimensional spatial network as a result of physical processes and chemical reactions [1,2,3]. The curing of elastomers leads to a loss of solubility and the elimination of the translation movements of macromolecules and a considerable reduction in the plastic deformation of elastomers.

For the vulcanization of elastomers, the presence of functional groups of defined chemical activity and chemical potential is necessary. Their chemical structure determines the capability of the suitable curing agent to the reaction with the speed being possible to regulate. In the most commonly used rubbers, there are double bonds >C=C<, α-methylene groups (e.g., present in natural rubber (NR), styrene-butadiene rubber (SBR), butadiene rubber (BR)), carboxyl groups –COOH (e.g., present in carboxylated acrylonitrile butadiene rubber (XNBR)), chlorosulfonic groups –SO_2_Cl and chlorine groups –Cl (in chlorosulfonated polyethylene (CSM)), there are bromine –Br or chlorine –Cl groups in brominated butyl rubber (BIIR), chlorinated butyl rubber (CIIR) and chloroprene rubber (CR)) and there are ethylene groups –CH_2_ or >CH– in saturated elastomers, as well as other groups in the main chain or in the side groups present in the special rubbers [4,5].

The effect of elastomer cross-linking is the chemical bonding of macromolecules by cross-links so that the resulting products are characterized by the properties of construction materials, among others such as high tensile strength, high tear strength, hardness, elasticity and resistance to aging and fatigue [5,6,7,8]. Furthermore, there is permanent deformation, friction coefficient and hysteresis loss decrease after the cross-linking, which in many cases is advantageous from the viewpoint of the final application of cured elastomers. The functional properties of cross-linked products depend on the type of elastomers, the type and amount of curing system used and the presence of fillers and plasticizers [9]. A conventional curing system—consisting of sulfur, selected activator and selected accelerator—is the most commonly used for the vulcanization of unsaturated diene rubbers [10,11,12,13]. The cross-linked elastomers are characterized by high mechanical strength, good abrasion resistance and high tear strength. A standard cross-linking system (with sulfur) is, however, not completely satisfactory as it is difficult to control the degree and homogeneity of cross-linking as well as the activation temperature. These latter aspects can lead to the undesirable scorching of the rubber composition due to early vulcanization and suboptimal sulfur dispersion, ending up with materials with poor hysteretic properties [14,15]. In addition, the use of a conventional curing system has disadvantages such as worse thermal resistance, low compression set, the migration of the curing agent or accelerators, the emission of toxic products of the accelerator decomposition (e.g., N-nitrosamines) and varied susceptibility of the elastomer to the pre-vulcanization and reversion [5,16].

The disadvantages of the conventional curing system and the numerous limits associated with their use have become stimuli for technologists to study new alternative methods of rubber cross-linking. Their idea is to use the advantages of the selected elastomers and simultaneously eliminate their defects by using unconventional curing agents or by suitable composition with other elastomers.

The production of unconventional elastomeric blends is possible during the chemical and physical modification of existing polymers [17,18,19]. The result of chemical modification is to change the chemical structure of macromolecules, leading to changes in their reactivity, and consequently to make significant changes in the properties of the resulting products. For example, this type of modification includes processes of esterification, etherification, dehalogenation, halogenation and elimination reactions of the polymers [20]. The advantage is the possibility of chemical modification to make significant changes in the construction of the polymers, and the downside is the need to conduct additional operations during the synthesis and the long and costly product testing. Properties of materials can also be modified with physical or physico-chemical processes, such as reactive processing including dynamic vulcanization and mechanochemical binding. An equally interesting and useful method is also manufacturing blends from two or more elastomers and making an elastomer functionalization.

The essence of the production of elastomeric blends is to take advantage of the individual components and eliminate their disadvantages. An additional aim is to improve the technological and processing properties of the basic elastomer under appropriate conditions and produce materials with interesting and controllable properties, different from the properties of vulcanizates of single elastomers cured in a conventional way. This can be achieved by the appropriate selection of the elastomers pair and the additional ingredients of the blend.

The purpose of our work was to determine the effects of zinc on the cross-linking characteristics, morphology, physical properties, fire resistance and toxicity of chloroprene and butadiene rubber (CR/BR) compounds unfilled and filled with unconventional fillers. In this paper, the CR/BR composites were investigated to determine the best natural silica fillers. Therefore, sillitin or chalcedonite as natural mineral fillers were used to increase their affinity to both applied elastomers (CR/BR) and to facilitate their application. For comparison, CR/BR blends were prepared with standard synthetic silica (aerosil). The research was based on the morphology characterization of the vulcanizates, the changes caused by the cross-linking with zinc, the cross-linking degree, swelling and mechanical and dynamic properties. The structure of the cured CR/BR blends was analyzed via scanning electron microscopy (SEM). The fire resistance studies and toxicity tests were performed using cone calorimetry or oxygen index methods.

## 2. Materials and Methods

### 2.1. Materials

Chloroprene rubber (CR, BAYPREN^®^216) with 40% bonded chlorine content and Mooney viscosity ML 1 + 4 (100 °C): 49 ± 5 was delivered by LANXESS GmbH, Köln, Germany. Butadiene rubber (BR, SYNTECA^®^44) with 97% *cis*-1.4 units and Mooney viscosity ML 1 + 4 (100 °C): 39 was delivered by Synthos S.A., Oswiecim, Poland. CR/BR blends were cross-linked with zinc (Zn). Zinc with a density of 7.13 g/cm^3^ (at 25 °C) and an average particle size of <150 µm was provided by Sigma-Aldrich Chemie, Steinheim am Albuch, Germany. Stearic acid from POCH S.A., Gliwice, Poland, was used as a dispersing agent (density of 0.94 g/cm^3^). In the tests, the following fillers were applied:

- Sillitin Z86 (Sil) from Hoffmann Mineral GmbH, Neuburg, Germany (pH = 8.5, an average particle size of 12.7 μm);
- Chalcedonite M12 (Chal) from Crusil Co, Inowlodz, Poland (pH = 6–8, an average particle size of 9 μm);
- Fumed silica Aerosil^®^380 (Aer) from Evonik Industries AG, Essen, Germany (pH = 3.7–4.5, an average particle size of 0.07 μm).

### 2.2. Formation of the CR/BR Blends and Its Cured Products

The tested blends containing chloroprene and butadiene rubbers were made with a laboratory two-roll mill (cylinders dimensions: 150 mm × 300 mm, friction ratio of 1:1.25, rolls temperature: 35 °C), David Bridge & Co, Rochdale, UK. The mixing time for the unfilled mix was 6 min, and for filled mixes it was 10 min, until all ingredients were well blended. First, the plasticization of raw elastomers (CR and BR) was carried out. The components were added as follows: CR and BR, a dispersing agent, a cross-linking agent and a filler. General formulations of the CR/BR mixes are specified in Table 1.

Various steel molds were used to vulcanize the tested blends. These molds were placed between the shelves of an electrically heated hydraulic press. The vulcanization of the CR/BR blends was carried out at a temperature of 160 °C, under a pressure of 200 bar and for the time determined on the basis of rheological curves (30 min).

### 2.3. Testing the Properties of the CR/BR Blends and Vulcanizates

#### 2.3.1. Cross-Linking Characteristics

The vulcanization kinetics of the tested compounds were determined using the oscillating disk rheometer (Alpha Technologies MDR 2000, Hudson, OH, USA) at 160 °C [21]. The essence of this process was to record the torque (M), dependent on the sample heating time (t), at a constant temperature (T), in the tested sample during its cross-linking. The value of the torque indicates the stiffness of the elastomer mix and changes with the progress of vulcanization. In this method, the following parameters were determined: optimal cure time as the time, when the torque reaches 90% of the increment (t_90_), scorch time as the safe processing time (t_02_), minimal torque (M_min_) and torque increment after 30 or 40 min of heating (∆M_30_, ∆M_40_) as the difference between the torque after heating and minimal torque values. The greater the torque increment, the greater the degree of vulcanization of the tested mix. The cure rate index (CRI), determining the rate of curing, was designated according to Formula (1):

(1) $$\mathrm{CRI}=\frac{100}{t_{90}-{\;t}_{02}}$$

Equilibrium swelling was designated in toluene or heptane. The samples after being weighed were placed in small bottles with toluene or heptane. The swelling phenomenon proceeded at 25 °C for 72 h (according to ASTM D 471 [22]). Next, the swollen samples were removed from the solvent, washed with diethyl ether, weighed, dried to constant weight at 50 °C and weighed again.

Equilibrium volume swelling (Q_v_) was determined using Formula (2):

(2) $$Q_{v}={\;Q}_{w}\times \frac{d_{v}}{d_{s}}$$

where Q_w_ is the value of the equilibrium mass swelling (mg/mg), d_v_ is the vulcanizate density (g/mL) and d_s_ is the solvent density (g/mL).

The degree of cross-linking (α_c_) was calculated using Formula (3):

(3) $$\alpha_{c}=\frac{1}{Q_{v}}$$

#### 2.3.2. Analysis of the Morphology of Vulcanizates

The surface morphology of the cured products was assessed using a scanning electron microscope (Hitachi Tabletop Microscope TM-1000, Tokyo, Japan). Before testing, the surfaces of the vulcanizates were gold-plated using the Cressington Sputter coater 108 auto vacuum sputtering machine (Redding, CA, USA) under a pressure of over 40 mbar for 60 s. The samples were cut with a scalpel to obtain their cross-sections. In this way, the transverse structure of the vulcanizate was examined. The samples prepared in this way were placed in the chamber of the scanning electron microscope and their morphology was tested.

#### 2.3.3. Mechanical Test

Measurements of mechanical properties were carried out according to ISO 37:2017, using the mechanical testing machine (Zwick 1435/Roell GmbH & Co. KG, Ulm, Germany) [23]. In this method, samples in the shape of paddles of the W2 type were used. Parameters determined from this test were as follows: (1) stress at elongation 100, 200, 300% (S_e100_, S_e200_, S_e300_), (2) tensile strength (TS_b_), (3) relative elongation at break (E_b_). Each determination was performed for five samples. The tensile rate of the samples was 500 mm/min.

Hardness (HA) was tested using the Zwick/Roell hardness tester according to ISO-48-4:2018. Samples had the shape of cylinders with a height of 6 mm. The hardness was calculated on the Shore A scale (°Sh A).

Measurements of the damping properties under compression conditions were carried out using the testing machine from Zwick Zmartpro 1435/Roell. The samples used in this study were in the shape of cylinders with dimensions of 35 mm in diameter, 17.8 mm in height. The maximum cyclic stress depended on the hardness of the vulcanizate (0.5 MPa, if HA = 30–40 °Sh A; 0.7 MPa, if HA = 41–50 °Sh A; 1.2 MPa, if HA = 51–65 °Sh A; 2.0 MPa, if HA = 66–85 °Sh A). The value of relative damping (T_τw_) was determined according to Formula (4):

(4) $$T_{\tau w}=\frac{{\Delta W}_{i}}{W_{\mathrm{ibel}}}*100$$

where ΔW_i_ is the difference between the work of compression and the work while reducing the strain (Nmm); W_ibel_ is work of compression (Nmm).

#### 2.3.4. Payne Effect

In order to study the dynamic properties of elastomers, the Payne effect (ΔG’) (Formula (5)) was calculated based on the storage modulus (G’):

(5) $$∆G\prime ={G\prime }_{\max}-{\;G\prime }_{\min}$$

where *G’*_max_ is the maximum value of the storage modulus (MPa), and *G’*_min_ is the minimum value of the storage modulus (MPa).

In this method, the loss modulus (G”) was also determined. For this purpose, the Ares G2 rotational rheometer (New Castle, UK) according to ISO 4664:2011 was applied. The samples of disc-shaped vulcanizates with a thickness of 2 mm and a diameter of 25 mm were used. The following parameters were used: soaking time of 10 s, an angular frequency of 10 rad/s, a logarithmic sweep with strain from 0.005 to 70% s, 20 points per decade and an initial force of 5 N. The measurement was carried out at 25 °C.

#### 2.3.5. Flammability Tests

Flammability of tested samples was determined with the use of the oxygen index method by means an apparatus built at the Institute of Polymer and Dye Technology of the Lodz University of Technology [24].

The test was performed according to PN-ISO 4589-2. The oxygen index (OI) was calculated using Formula (6) [25]:

(6) $$\mathrm{OI}=\frac{O_{2}}{O_{2}+N_{2}}\times 100\%$$

A cone calorimeter (Fire Testing Technology Ltd., East Grinstead, UK) was used to evaluate the flammability of investigated samples. Squared specimens with dimensions of 100 × 100 × 2 mm were irradiated horizontally using a 35 kW/m^2^ heat flux.

Based on the obtained results, the fire hazard was calculated as the fire propagation rate (1/t_flashover_) using Formula (7) [26]:

(7) $$\frac{1}{t_{\mathrm{flashover}}}=\frac{{\mathrm{HRR}}_{\mathrm{MAX}}}{\mathrm{TTI}}$$

where HRR_max_ is the maximum heat release rate, TTI is the time to ignition.

#### 2.3.6. Toxicity

**Figure 1:**
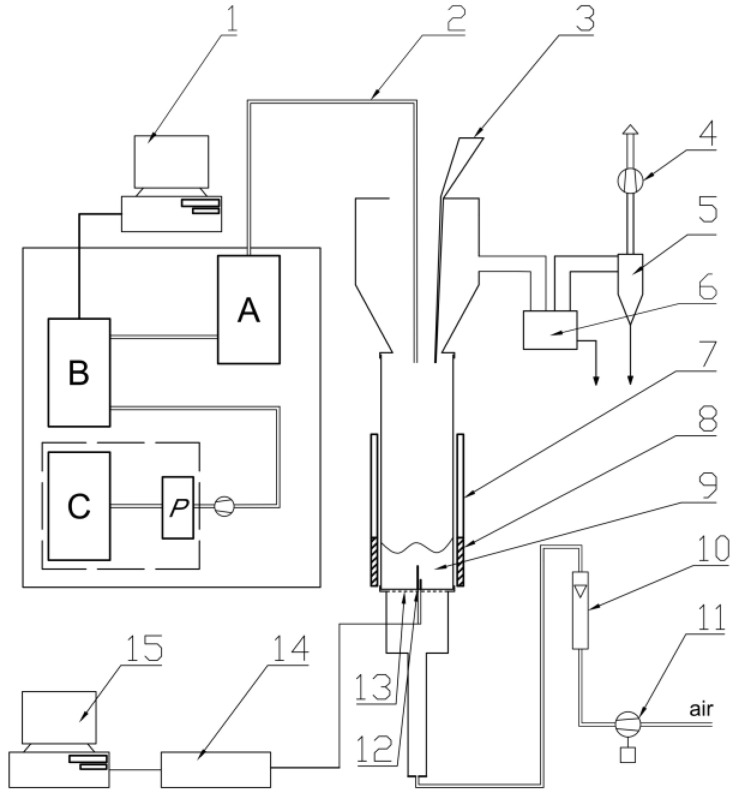
Scheme of the fluidized bed reactor: 1—computer connected with the FTIR analyzer; 2—heated probes for sampling the flue gases; 3—batcher; 4—exhaust fan; 5—cyclone; 6—ash trap for coarser particles; 7—movable radiation shield; 8—heating jacket; 9—bubbling bed; 10—air rotameter; 11—blower for fluidizing air; 12—two thermocouples; 13—flat, perforated, metal plate distributor; 14—A/D convertor for thermocouple signals; 15—computer storing chemical analysis quantities and temperature; A—mobile conditioning system of Gasmet DX-4000, B—analyzer FTIR (Gasmet DX-4000), C—Horiba PG250, P—Peltier’s cooler [27,28].

The gaseous components formed during the thermal decomposition of the test samples were analyzed qualitatively and quantitatively. In the presented method, using the fluidized bed technique, a laboratory station was used (Figure 1) [27]. A quartz fluidized bed reactor with a length of 500 mm, an outer diameter of 100 mm and a wall thickness of approximately 2 mm was an essential part of the set-up. Air was the fluidizing agent, which provided the oxidizing conditions in the reactor. The air flow rate was approximately 15 L/min and was controlled using a TSI 40241 flow meter. Air was supplied from the air fan through a distributor (perforated plate). Two hundred grams of aluminosilicate microspheres with a particle diameter of 160–180 um was used as the fluidized bed. Analysis of the gaseous components was performed at three temperatures: 450 °C, 550 °C and 750 °C [27,28]. A resistive heating jacket connected to an autotransformer was used to heat and stabilize the temperature of the bed. Ni-NiCr thermocouples placed in the reactor 20 and 50 mm from the distributor were used to monitor the temperature of the bed. The masses of the polymer composite samples were selected experimentally. A composite sample weighing between 0.01 and 0.11 g was dispensed into the bed, with three samples of each composite type analyzed. The concentrations of the resulting gases during thermal decomposition were monitored continuously; hence, when the concentrations of CO_2_, H_2_O and CO reached background levels, another sample was placed in the reactor [27,28].

An FTIR analyzer, the Gasmet DX-4000, was used to obtain measurement data. Using Fourier transform infrared spectroscopy (FTIR), the instrument incorporating a Michaelson interferometer allows gas spectra to be obtained. Based on a library of reference spectra, the software deconvolved the resulting spectra and thus determined the concentrations of compounds present in the thermal decomposition products. A gas analyzer, the Horiba PG250, was used to control measurements of CO, CO_2_ and SO_2_ using a non-dispersive infrared (NDIR) measurement method [27,28,29].

In this method, the results are given as toxicometric indicators (W_LC50,_ W_LC50M_, W_LC50SM_) in specific emission units (E, g/g). W_LC50_ index is the maximum toxic concentration of gaseous product formed during the thermal decomposition and combustion of testing material in the temperature (T), according to Formula (8) [27]:

(8) $$W_{\mathrm{LC}50}=\frac{{\mathrm{LC}}_{50}^{30}}{E}\;$$

where E is the emission (g/g), and LC_50_^30^ is the lethal concentration of gaseous product of thermal decomposition and combustion of testing material which causes a 50% lethality of testing animals during 30 min exposure (g/m^3^).

W_LC50M_ index is defined as the resultant of the W_LC50_ index of individual products of thermal decomposition and combustion for a given temperature, according to Formula (9):

(9) $$\frac{1}{W_{\mathrm{LC}50M}}=\overset{n}{\sum }\frac{1}{W_{\mathrm{LC}50}}\;$$

where n is the number of gaseous products.

W_LC50SM_ index is defined as the arithmetic average of W_LC50M_ indices, according to Formula (10):

(10) $$W_{\mathrm{LC}50\mathrm{SM}\;}=\frac{W_{\mathrm{LC}50M450}+{\;W}_{\mathrm{LC}50M550}+{\;W}_{\mathrm{LC}50M750}}{3}\;$$

## 3. Results and Discussion

### 3.1. Cross-Linking Results of Unfilled and Filled CR/BR/Zn Blends

The cross-linking process is connected with two phenomena: physical heat transfer and chemical reaction [30,31]. The rubber vulcanization creates cross-links between the polymer chains, generally with the release of energy that changes the properties of the final material. Rheologically, as new cross-links are formed, the shear modulus of the elastomer increases. Therefore, the rheology of rubbers provides information on the degree of cross-linking and on optimal cross-linking parameters, such as cure temperature and cure time, as these are influential variables in the vulcanization processes. Therefore, the determination of cross-linking characteristics is very important. In terms of vulcametric results, the CR/BR composites cured with zinc were characterized by a long scorch time (t_90_ = 5.54 min). The incorporation of sillitin or chalcedonite significantly shortened the t_90_ parameter (to values of 4.38 min and 4.42 min, respectively). The shortest scorch time (t_02_ = 1.10 min) and the shortest cure time (29.18 min) were found for the CR/BR/Zn blend filled with aerosil. The longest cure time (t_90_ = 31.36 min) was observed for the unfilled mix. It is worth noting that the presence of fillers in the CR/BR/Zn blends has little effect on the vulcanization time (Table 2).

The presence of fillers clearly changed the minimal vulcametric torque. The M_min_ value of the unfilled CR/BR/Zn blend was 0.55 dN·m. The application of sillitin or chalcedonite as a filler led to an increase in minimal torque to values of 0.74 dN·m and 0.70 dN·m. The highest minimal torque was recorded for the blend filled with aerosil (4.81 dN·m). Such a large increase in the M_min_ parameter results from the high activity of aerosil and the large specific surface area of this filler (BET of aerosil = 380 m^2^/g). Less pronounced differences in torque increment were observed after 30 min of heating (∆M_30_) between unfilled and filled blends. For blends without a filler and filled with chalcedonite, the ∆M_30_ parameter was comparable (~6.48 dN·m). The filling tested blend with sillitin led to less of a torque increment (5.86 dN·m), but the addition of aerosil caused the highest (above 40%) torque increment, equal to 8.79 dN·m. Such a significant increase in the torque increment for aerosil-containing blends compared to the other samples filled with sillitine or chalcedonite may result from the formation of additional bonds between the chloroprene rubber and aerosil. As a result of the chemical reaction between the silanol groups on the surface of this filler and the allyl chlorine atom in the chloroprene rubber, new bonds can be formed, leading to an increase in the degree of cross-linking. When analyzing the obtained vulcametric results, 160 °C was selected as the cure temperature and 30 min was selected as the cure time for all CR/BR/Zn composites.

Swelling studies were conducted to further analyze the degree of cross-linking of CR/BR/Zn compounds. The degree of swelling is one of the basic parameters determining the resistance of vulcanizates to solvents (in this case: toluene). The results showed that the presence of fillers did not affect the degree of swelling (Table 2). The swelling of both unfilled and filled CR/BR/Zn vulcanizates was comparable (from 8.15 mL/mL to 9.21 mL/mL). The degree of cross-linking calculated on the basis of the equilibrium swelling results was in the range of 0.11 (for sillitin application) to 0.13 (for aerosil application). The obtained swelling results were reflected in the mechanical properties: among the filled CR/BR/Zn variants, the product with the lowest degree of swelling and thus the highest degree of cross-linking showed the highest tensile strength (Table 3).

### 3.2. Mechanical and Dynamic Properties of Unfilled and Filled CR/BR/Zn Vulcanizates

The mechanical and dynamic properties of rubber materials are directly related to the cross-linking parameters [31,32]. The unfilled and filled CR/BR/Zn vulcanizates were tested for hardness, damping, stress at 100, 200, and 300% strains, tensile strength, elongation at break, storage modulus and loss modulus. Test results are presented in Table 3. The high tensile strength is required for rubber products applied and used in various areas.

The application of different filling methods of the CR/BR/Zn composites highlighted the influence of the filler type (sillitin, chalcedonite, or aerosil) on their mechanical properties. Unfilled vulcanizates were characterized by the worst mechanical properties, especially with the stress at the 100% strain (0.66 ± 0.01 MPa) and tensile strength (8.56 ± 0.41 MPa). The incorporation of sillitin or chalcedonite improved the tensile strength to equal to ~8.97 MPa. The highest TS_b_ (10.97 ± 0.72 MPa) was achieved after the filling of the CR/BR/Zn vulcanizate with aerosil. This result is quite obvious as aerosil is the most active filler among those used in these studies. The surface area for aerosil is several times higher than BET for other fillers and amounts to 380 m^2^/g. Surface area is probably the most important morphological characterization of reinforcing fillers because it corresponds to the extension of the interface, i.e., the interaction between elastomer and filler surface. Compared to other used fillers, aerosil gives a higher elastomer–filler interaction. The result is higher tensile strength. Elastomer compounds’ reinforcement by aerosil can be generally considered as the consequence of the adsorption of rubber chains onto the surface of this filler. In addition, we have observed that both unfilled vulcanizate and vulcanizate filled with sillitin and chalcedonite had the comparable elongation at break (~755%), whereas the samples containing aerosil showed the E_b_ value equal to 647% (Table 3). This fact may indicate lower elasticity and greater hardness of aerosil-containing vulcanizates.

Shore hardness (HA) depends on the elastic properties of the tested material (Young’s modulus). The hardness of materials is a property that allows one to determine changes which occur from the surface to the depth of the material. By analyzing the hardness of the obtained compositions, it was found that the incorporation of aerosil into the CR/BR/Zn samples led to the production of vulcanizates with the highest hardness (65.3 °ShA). Such a high hardness of samples containing aerosil was due to the highest degree of cross-linking achieved by them (Table 3). The well-developed surface area of aerosil and the possibility of chemical reactions occurring at elevated temperatures between the silanol groups present on the aerosil surface and the chlorine atom in CR macromolecules contribute to the increase in the cross-linking degree and thus to a greater hardness of the CR/BR vulcanizates.

The above-mentioned dependence is also the cause of the highest damping relative (T_τw_) recorded for the sample filled with aerosil. The T_τw_ value of the CR/BR/Zn vulcanizate was 10.25%, but the application of the filler improved the damping properties (the T_τw_ values were 19.88%, 21.27% and 29.40% for sillitin, chalcedonite and aerosil, respectively). It is worth noting that the higher the damping relative, the more the vulcanizate is able to minimize vibrations (Table 3).

Table 3 also presents the results of testing the dynamic properties. Dynamic studies of CR/BR/Zn vulcanizates were carried out at constant temperature, with constant deformation frequency and changing deformation amplitude. Under these conditions, the storage modulus decreases as the deformation amplitude increases because the interactions, e.g., rubber–filler or filler–filler, are destroyed. This phenomenon is called the Payne effect. The aerosil-filled vulcanizate obtained the highest Payne effect (ΔG’ = 0.392 MPa), which most likely results from the formation of an extra network by aerosil particles and their tendency to form agglomerates or aggregates. The size distribution of aggregates is a very important structural feature of active fillers. In addition, the quality of the molecular filler dispersion in the rubber is a factor that strongly affects the mechanical and dynamic properties of the final products. Dispersibility is important for obtaining aerosil-reinforced vulcanizates. Obviously, the dispersibility of any filler depends mainly on the interactions between aggregates and/or agglomerates. In the case of aerosil, these interactions are mainly due to the hydrogen bonds that exist between the silica grains. This is indicated by the maximum storage modulus, which is higher the greater the specific surface area of the filler (BET of aerosil = 380 m^2^/g). For the samples containing chalcedonite or sillitin, the Payne effect was clearly lower (0.193 and 0.185 MPa, respectively) and comparable to the ΔG’ value of the unfilled compound. The slight Payne effect in these products may indicate weak filler–filler or filler–rubber interactions, which may be due to the small specific surface area (the BET of chalcedonite and sillitin is equal to 10 and 12 m^2^/g, respectively).

The loss modulus (G”) informs one about the amount of energy dissipated during the dynamic deformation of the rubber product and its transformation into heat. Its value depends on the destruction and rebuilding of the network structure that the filler can create. The G” modulus is higher the more the filler networks are destroyed and rebuilt during one deformation cycle. The highest value of the loss modulus (G”_max_ = 0.114 MPa) was obtained for the CR/BR/Zn/aerosil, which confirms the possibility of the formation of the filler network in the elastomeric matrix. The smallest value of the maximum loss modulus (G”_max_~0.044 MPa) was obtained for the unfilled sample and the vulcanizate containing sillitin. Extra network formation by the filler in the elastomers or strong filler–filler or filler–rubber interactions determine to a large extent the morphology, degree of cross-linking and mechanical–dynamic properties of CR/BR/Zn vulcanizates.

### 3.3. SEM Analysis of CR/BR/Zn Surface Morphology

**Figure 2:**
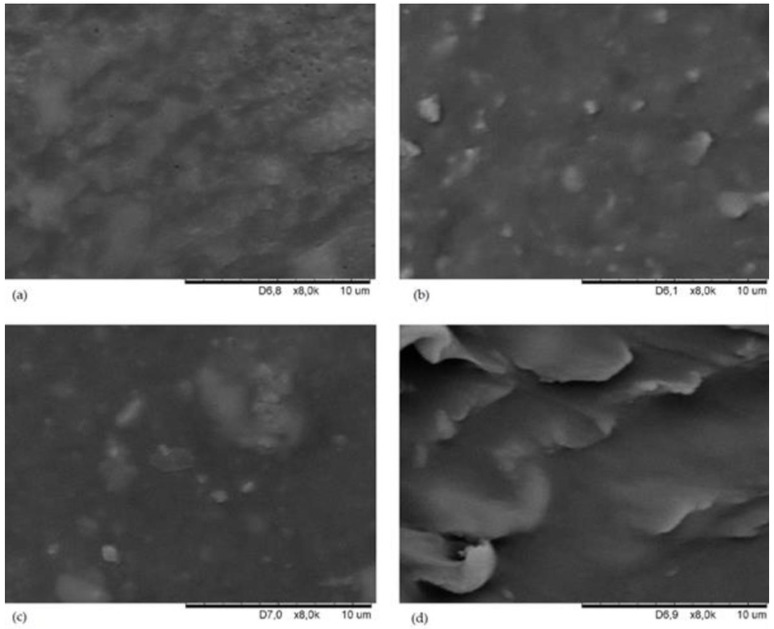
SEM images of the surface of CR/BR/Zn (**a**); CR/BR/Zn/sillitin (**b**); CR/BR/Zn/chalcedonite (**c**); and CR/BR/Zn/aerosil (**d**).

The aim of SEM tests was to determine the effect of applied fillers on the morphology of the obtained CR/BR/Zn compounds. The surface morphology of composites containing sillitin, chalcedonite or aerosil at a 8 k magnification are shown in Figure 2.

In the SEM image of the CR/BR/Zn vulcanizate, two mutually interpenetrating phases were visible (Figure 2a). It was a rather parallel arrangement of phases. The SEM image of the CR/BR/Zn sample filled with sillitin showed brighter areas in the elastomeric matrix, indicating small aggregates of this filler (Figure 2b). In the structure of the CR/BR/Zn/chalcedonite vulcanizate, both individual filler particles as well as their aggregates and agglomerates (especially one agglomerate in the central part of the SEM image) were visible (Figure 2c). The sillitin and the chalcedonite have similar specific surface areas (BET of sillitin = 12 m^2^/g, BET of chalcedonite = 10 m^2^/g) and a similar particle size equal to 9 and 13 μm, respectively, whereas in the SEM image of the CR/BR/Zn/aerosil composition, large flat agglomerates of silica forming a layered system of the elastomer-filler composition were observed (Figure 2d). This is probably connected with the most developed specific surface area of this filler (BET of aerosil = 380 m^2^/g) and the smallest size of grains prone to forming large clusters in the elastomeric matrix. The presence of large agglomerates in this vulcanizate confirm the highest Payne effect, as mentioned earlier (Table 3, Section 3.2). It is worth noting that the dispersion of the filler is primarily influenced by the strength of interactions between aggregates and/or agglomerates. This magnitude of interactions results directly from the surface energy of the selected filler. Another factor determining the correct or incorrect dispersion of the filler is its morphology and surface area. The smaller the surface area, the better the dispersion.

**Figure 3:**
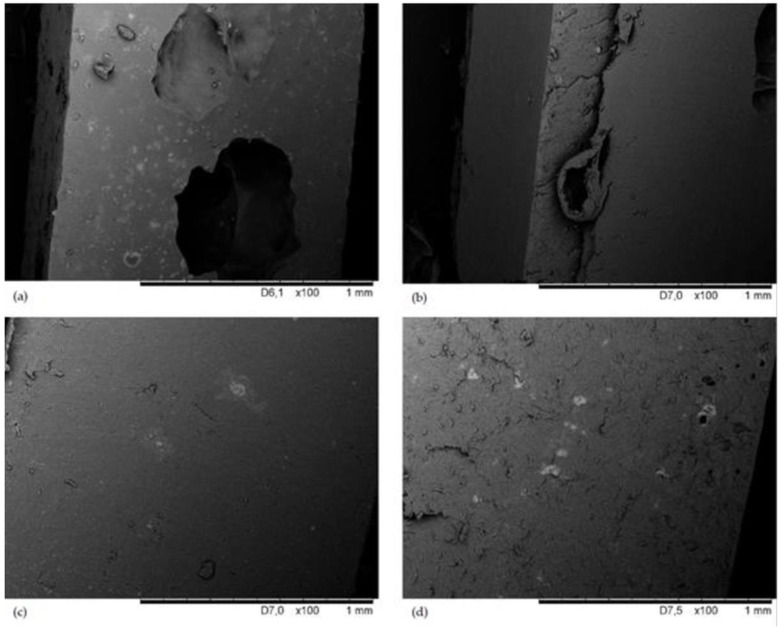
SEM images of the cross-section of CR/BR/Zn (**a**); CR/BR/Zn/sillitin (**b**); CR/BR/Zn/chalcedonite (**c**); and CR/BR/Zn/aerosil (**d**).

The cross-section surface morphologies of the obtained vulcanizates are shown in Figure 3. The cross-section morphology of the unfilled vulcanizate (Figure 3a) contained large empty spaces, which proves unsatisfactory processing of these composites. The application of the filler changed the cross-section of the resulting vulcanizates. The presence of sillitin in the CR/BR vulcanizate resulted in a rough morphology with frequent grooves (Figure 3b). Figure 3c,d shows the smooth cross-section of vulcanizates filled with chalcedonite or aerosil, although the morphology of the CR/BR/Zn/aerosil was cracked.

### 3.4. Flammability, Fire Hazard and Toxicity of Unfilled and Filled CR/BR/Zn Vulcanizates

Insufficient thermal stability during ordinary use, undesirable flammability and too-high fire hazard are very important problems for rubber products. The thermal properties of elastomeric materials depend on their chemical structure, the degree of cross-linking and their compounds. Standard cross-linked butadiene rubber (i.e., with sulfur in the presence of accelerators and activators) is a flammable product, as determined by the oxygen index (OI) method. The OI value of BR vulcanizates is only 17%. On the other hand, chloroprene rubber cross-linked with zinc oxide and magnesium oxide has a much higher oxygen index (OI = 26%), which makes it a flame-retardant product. It was found that the combination of both elastomers and their cross-linking with zinc led to the materials with lower flammability (OI = 30.1%). The filling of the CR/BR/Zn blends containing sillitin, chalcedonite or aerosil caused the oxygen index to be 37.5%, which classifies them as non-flammable rubber products (Table 4). The reason for such a high value of the oxygen index in the tested CR/BR vulcanizates is probably due to the inter-elastomeric reactions that take place during the unconventional cross-linking of such compositions with zinc.

**Figure 4:**
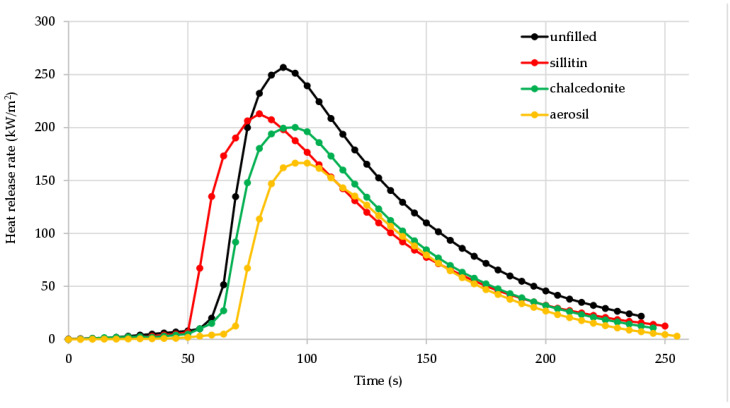
Heat release rate (HRR) of unfilled or filled vulcanizates.

**Figure 5:**
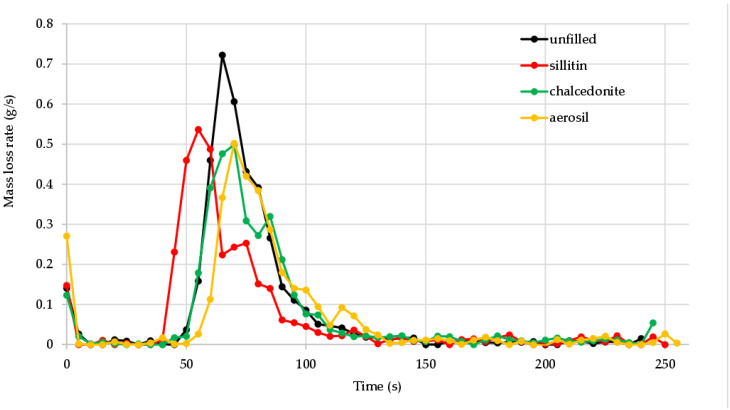
Mass loss rate (MLR) of unfilled or filled vulcanizates.

The oxygen index method is not a sufficient test to assess the flammability of elastomers, as it can only be used to compare this phenomenon. Thus, cone calorimetry was performed for a detailed analysis of flammability parameters. On the basis of this method, the basic properties determining the fire hazard were determined, namely, time to ignition (TTI), total heat release (THR), heat release rate (HRR), time to maximum heat release rate (tHRR_max_), total heat release (THR), effective heat of combustion (EHC), mass loss rate (MLR) and average mass loss rate (AMLR) [26]. The data contained in Table 4 clearly indicate that CR/BR/Zn vulcanizates are materials with high resistance to burning. The average maximum heat release (HRR_max_) of the unfilled vulcanizates was only 256.79 kW/m^2^. For the sample filled with aerosil, the HRR_max_ value decreased to 166.46 kW/m^2^. The presence of sillitin or chalcedonite in the tested compounds led to the HRR_max_ values being equal to 212.89 or 200.20 kW/m^2^, respectively (Figure 4). Sillitin, similarly to chalcedonite as well as aerosil, decrease the fire hazard of the studied composites. In the case of composites containing sillitin, the HRR parameter is a little higher than in the case of composites containing chalcedonite or silica. In our opinion, the boundary layer created during the combustion of composites containing chalcedonite or silica has better isolating properties and is more homogenous than in the case of composites containing sillitin. The homogenous structure and isolating properties of the boundary layer are directly connected with the size of the filler. The average size of sillitin grain is 12.7 um, whereas for chalcedonite and silica it is, respectively, 9 and 0.07 um. It is worth noting that the heat release rate (HRR) during the combustion of all CR/BR/Zn vulcanizates was incomparably low compared to other rubber materials. For comparison, cross-linked acrylonitrile butadiene rubber (NBR18, where the number 18 indicates the weight percentage of bound acrylonitrile) was characterized by the HRR value being equal to 3569.23 kW/m^2^ [33]. The average mass loss rate (AMLR) of the tested compounds, which proves the dynamics of material combustion dynamics, reached the highest value (53.90 g/m^2^·s) in the case of the unfilled vulcanizate, but the presence of aerosil resulted in the reduction in the AMLR value to 32.57 g/m^2^·s. Figure 5 shows the mass loss rate (MLR) versus the incineration time of unfilled and filled tested vulcanization. It is clearly visible that the mass loss rate was much smaller for the filled samples, which indicates the positive effect of all the fillers used.

The cone calorimetry results allowed one to calculate the fire hazard related to the fire propagation rate (i.e., 1/t_flashover_). This is defined as the time inverse to the flashover effect [34]. The least fire risk was found for the composites filled with aerosil (1/t_flashover_ equal to 2.73 kW/m^2^·s) or chalcedonite (1/t_flashover_ equal to 3.85 kW/m^2^·s). The vulcanizate filled with sillitin had the greatest fire risk (1/t_flashover_ equal to 5.46 kW/m^2^·s) (Table 4). It should be stressed that the fire hazard determined for the tested CR/BR vulcanizates was clearly lower than the fire risk of products made of acrylonitrile butadiene rubber, styrene-butadiene rubber and butadiene rubber, whose corresponding values amounted to 66.06, 31.68 and 56.37 kW/m^2^·s, respectively [25]. In addition, the tested CR/BR/Zn vulcanizates pose a significantly lower fire hazard than that of the popularly used polyethylene or polypropylene (1/t_flashover_ equal to 20.15 and 44.22 kW/m^2^·s, respectively). Thus, the obtained results confirm that the all zinc-cured CR/BR products are non-flammable and pose a low fire hazard. The FIGRA parameter showing the ratio of maximum heat release rate to time to maximum heat release rate was similar. The highest FIGRA index was achieved for the sample without fillers (2.85 kW/m^2^·s), and the lowest was for the vulcanizate filled with aerosil (1.75 kW/m^2^·s). The maximum average heat release rate (MARHE) of the CR/BR/Zn/aerosil vulcanizate was 37% lower (MARHE = 67.1 kW/m^2^) than for the unfilled sample (MARHE = 106.1 kW/m^2^). The analysis of the obtained results confirms that the use of aerosil as a filler has the greatest impact on reducing the fire hazard and favors the formation of a thermally stable boundary layer that hinders the mass and energy flow between the flame and the sample.

In addition to the resistance to burning, the toxicity of substances released as a result of the combustion of elastomeric materials is also important. The emissions of carbon(IV) oxide (CO_2_), carbon(II) oxide (CO) and other gaseous substances such as nitrogen(IV) oxide (NO_2_), sulfur(IV) oxide (SO_2_), hydrogen chloride (HCl) and hydrogen cyanide (HCN) are the main causes of product toxicity resulting from thermal decomposition and the combustion of tested vulcanizates. The parameter that takes into account the concentrations of all six gases emitted at temperatures T = 450, 550 and 750 °C is known as the toxicometric index (W_LC50SM_), described by Formula (10) [25].

Table 5 shows the specific emission of selected gaseous products formed during the combustion and decomposition of tested vulcanizates at the temperatures of 450, 550 and 750 °C. The presented data show that the highest emission values were observed, regardless of the decomposition temperature and the sample type, for carbon(IV) oxide and carbon(II) oxide. The carbon(IV) oxide emission for filled vulcanizates was noticeably lower than in the case of the unfilled CR/BR/Zn vulcanizate. Undoubtedly, the value of the toxicometric index was influenced by the emission of SO_2_ (0.01 g/g for all samples) and HCl (the highest emission for an unfilled sample: 0.15–0.20 g/g). A potential source of HCl was chlorine bound to macromolecules of the chloroprene rubber.

**Figure 6:**
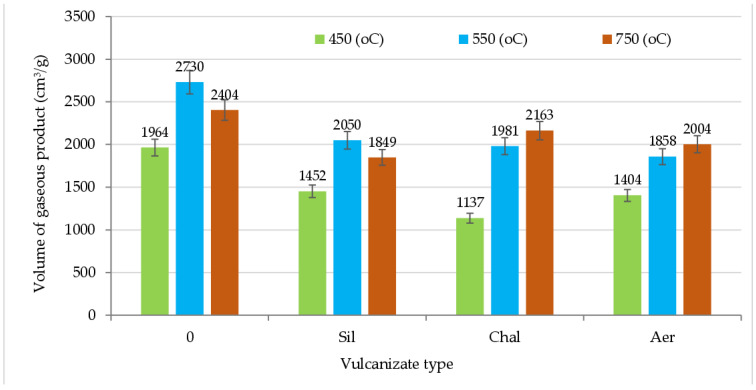
The volume of gaseous products formed during the combustion and decomposition of unfilled and filled vulcanizates at the temperatures of 450, 550 and 750 °C.

The results presented in Figure 6 show that the volume of gases generated during the thermal decomposition of CR/BR/Zn vulcanizates, in the temperature range of 450, 550 and 750 °C, was significantly higher for the unfilled product and was the lowest for the sample containing chalcedonite. For example, at 450 °C, the volume of gases emitted during the decomposition of CR/BR/Zn/chalcedonite was 42% lower than for the unfilled vulcanizate and ~20% lower than for the vulcanizate filled with aerosil or sillitin.

The basis for the classification of thermal decomposition and combustion products is the parameter W_LC50SM_. According to PN-B-02855:1988, the products should be considered very toxic if W_LC50SM_ ≤ 15, the products are toxic if 15 ≤ W_LC50SM_ ≤ 40 and the products are moderately toxic if W_LC50SM_ > 40. The WLC_50SM_ values show that gaseous products generated during the decomposition of CR/BR vulcanizates should be considered as very toxic (Table 6). The applied fillers slightly increased the toxicity of gaseous products formed as a result of thermal decomposition and combustion of the tested elastomeric materials, determined by the W_LC50SM_ index. It should be remembered that the reactions taking place on the surface of the combusted materials play an important role in the combustion process of elastomers, because they affect both the processes occurring in the flame and the processes of thermal decomposition of the elastomer in the solid phase. The formation of gaseous products, accelerating combustion processes, occurs mainly as the result of the polymer decomposition. In addition to the conduction, convection and radiation, strongly exothermic oxidation reactions in the surface layer between the solid and gas phases are the significant sources of thermal energy necessary to support the processes of elastomer decomposition. Limiting the oxidation processes in the surface layer of the tested vulcanizates may contribute to the extinction of the flame.

## 4. Conclusions

Elastomeric compounds including chloroprene and butadiene rubbers (CR/BR) can be efficiently vulcanized with zinc. The use of zinc for the cross-linking of the CR/BR blends and the application of sillitin, chalcedonite or aerosil as a filler led to elastomeric products ensuring the satisfaction of physical, dynamical and mechanical properties and high fire resistance.

An analysis of SEM images confirmed that the curing agent (zinc) as well as the filler type (sillitin, chalcedonite, aerosil) affected the surface morphology of the CR/BR vulcanizates. The use of zinc and chalcedonite led to a less rough structure.

The best results were obtained for the CR/BR/Zn composites containing aerosil as a filler. It was observed that the presence of this filler led to the highest cross-linking degree (α_c_ = 0.13, ΔM_30_ = 9.19 dNm), the best mechanical properties (TS_b_ = 10.97 MPa, HA = 65.3 °ShA, T_τw_ = 29.40%) and the best dynamic properties (G’_max_ = 0.423 MPa, G”_max_ = 0.114 MPa, ΔG’ = 0.392 MPa). This was also reflected in SEM studies. The CR/BR/Zn vulcanizates containing other fillers (sillitin or chalcedonite) were characterized by better properties than unfilled products, proving that the presence of studied fillers enables the creation of cross-bonds in the elastomeric matrix.

The flammability of unfilled and filled CR/BR/Zn products was designated. The properties characterizing the behavior of the tested vulcanizates under fire conditions were determined using the oxygen index and cone calorimetry methods. The obtained results showed that all tested CR/BR/Zn vulcanizates pose a low fire hazard and can be classified as non-combustible materials. In addition, it was confirmed that among all of the tested fillers, the presence of aerosil in CR/BR/Zn compositions clearly reduces the fire hazard expressed by the maximum heat release rate (HRR_max_ = 166.46 kW/m^2^), total heat released (THR = 10.0 MJ/m^2^) or mass loss rate (MLR = 0.154 g/s). The highest FIGRA index was achieved for the sample without fillers (2.85 kW/m^2^·s), and the lowest was for the vulcanizate filled with aerosil (1.75 kW/m^2^·s). The maximum average heat release rate (MARHE) of the CR/BR/Zn/aerosil vulcanizate was 37% lower (MARHE = 67.1 kW/m^2^>) than for the unfilled sample (MARHE = 106.1 kW/m^2^). The analysis of the obtained results proves that the use of aerosil as a filler has the greatest impact on reducing the fire hazard and favors the formation of a thermally stable boundary layer that hinders the mass and energy flow between the flame and the sample.

The obtained results clearly indicate that the zinc-cured CR/BR products, both unfilled and filled with aerosil, sillitin or chalcedonite, belong to non-flammable materials and show a low fire hazard (1/t_flashover_ = 2.73–5.46 kW/m^2^∙s). However, gaseous products generated during the decomposition of CR/BR vulcanizates should be considered as very toxic (WLC_50SM_ = 4.15–5.16).

The analysis of our research results suggests that they should be continued in the near future. An interesting issue will be the surface modification of silica particles with silanes and the study of nanoflower-like silica morphology.

## Figures and Tables

**Table 1:** Formulations of the CR/BR compounds.

Ingredient	Ingredient Content (phr)			
CR/BR	80/20	80/20	80/20	80/20
Stearic acid	1	1	1	1
Zn	2.5	2.5	2.5	2.5
Sillitin (Sil)	-	30	-	-
Chalcedonite (Chal)	-	-	30	-
Aerosil (Aer)	-	-	-	30

phr—parts per hundred of rubber.

**Table 2:** The cross-linking characteristic of the unfilled or filled composites cured at 160 °C.

Parameter	Filler			
	-	Sil	Chal	Aer
t_02_ (min)	5.54	4.38	4.42	1.10
t_90_ (min)	31.36	29.26	29.19	29.18
M_min_ (dN·m)	0.55	0.74	0.70	4.81
∆M_30_ (dN·m)	6.25	5.86	6.70	9.19
CRI (min^−1^)	3.87	4.02	4.04	3.56
Q_v_ (mL/mL)	8.48	9.21	8.33	8.15
α_c_	0.12	0.11	0.12	0.13

t_02_—scorch time, t_90_—cure time, M_min_—minimal torque, ∆M_30_—torque increment after 30 min of heating, CRI—cure rate index, Q_v_—equilibrium swelling degree in toluene, α_c_—cross-linking degree.

**Table 3:** The mechanical–dynamical properties of the unfilled or filled vulcanizates, T = 160 °C, t = 30 min.

Parameter	Filler			
	-	Sil	Chal	Aer
S_e100_ (MPa)	0.66 ± 0.01	0.81 ± 0.02	0.75 ± 0.03	2.72 ± 0.18
S_e200_ (MPa)	1.02 ± 0.02	1.14 ± 0.03	1.10 ± 0.07	4.04 ± 0.24
S_e300_ (MPa)	1.41 ± 0.04	1.50 ± 0.03	1.45 ± 0.11	5.23 ± 0.28
TS_b_ (MPa)	8.56 ± 0.41	8.94 ± 0.33	9.00 ± 0.48	10.97 ± 0.72
E_b_ (%)	750 ± 50	762 ± 44	756 ± 70	647 ± 69
HA (°Sh A)	30.4 ± 1.2	39.1 ± 0.8	35.7 ± 0.5	65.3 ± 0.9
T_τw_ (%)	10.25 ± 0.13	19.88 ± 0.09	21.27 ± 2.43	29.40 ± 1.07
G’_max_ (MPa)	0.215	0.223	0.268	0.423
G”_max_ (MPa)	0.041	0.047	0.098	0.114
ΔG’ (MPa)	0.182	0.185	0.193	0.392

S_e100_, S_e200_, S_e300_—stress at 100%, 200%, 300% strain; TS_b_—tensile strength; E_b_—elongation at break; HA—hardness; T_τw_—damping relative; G’_max_—maximum storage modulus; G”_max_—maximum loss modulus; ΔG’—Payne effect.

**Table 4:** Parameters characterizing the flammability and fire hazard of the unfilled or filled vulcanizates, determined by oxygen index and cone calorimetry.

Parameter	Filler			
	-	Sil	Chal	Aer
t_b_ (s)	5	5	5	5
OI (%)	30.1	37.5	37.5	37.5
TTI (s)	55	39	52	61
THR (MJ/m^2^)	17.8	13.6	12.5	10.0
HRR (kW/m^2^)	150.73	132.81	130.33	117.06
HRR_max_ (kW/m^2^)	256.79	212.89	200.20	166.46
tHRR_max_ (s)	90	80	95	95
THR (MJ/m^2^)	17.8	13.6	12.5	10.0
EHC (MJ/kg)	8.92	8.06	7.17	6.59
EHC_max_ (MJ/kg)	79.76	72.01	66.15	71.77
MLR (g/s)	0.149	0.146	0.159	0.154
MLR_max_ (g/s)	0.722	0.536	0.498	0.502
AMLR (g/m^2^·s)	53.90	39.44	40.29	32.57
1/t_flashover_ (kW/m^2^·s)	4.67	5.46	3.85	2.73
FIGRA (kW/m^2^·s)	2.85	2.66	2.10	1.75
MARHE (kW/m^2^)	106.1	97.5	83.6	67.1

t_b_—burning time, OI—oxygen index, TTI—time to ignition, THR—total heat release, HRR—heat release rate, HRR_max_—maximum heat release rate, tHRR_max_—time to maximum heat release rate, THR—total heat release, EHC—effective heat of combustion, EHC_max_—max effective heat of combustion, MLR_max_—maximum mass loss rate, AMLR—average mass loss rate, 1/t_flashover_—fire propagation rate, FIGRA = HRR_max_/tHRR_max_, MARHE—maximum average heat release rate.

**Table 5:** Specific emission of selected, in accordance with the standard, gaseous products formed during the decomposition and combustion of unfilled and filled vulcanizates at the temperatures of 450, 550 and 750 °C.

Filler	T (°C)	Emission (g/g)					
		CO_2_	CO	NO_2_	SO_2_	HCl	HCN
-	450	1.88 ± 0.16	0.27 ± 0.01	0.00 ± 0.00	0.01 ± 0.00	0.15 ± 0.04	0.00 ± 0.00
	550	2.49 ± 0.27	0.40 ± 0.08	0.00 ± 0.00	0.01 ± 0.00	0.20 ± 0.02	0.00 ± 0.00
	750	2.84 ± 0.23	0.01 ± 0.01	0.00 ± 0.00	0.01 ± 0.00	0.16 ± 0.01	0.00 ± 0.00
Sil	450	1.28 ± 0.20	0.26 ± 0.02	0.00 ± 0.00	0.01 ± 0.00	0.13 ± 0.03	0.00 ± 0.00
	550	1.78 ± 0.17	0.36 ± 0.01	0.00 ± 0.00	0.01 ± 0.00	0.14 ± 0.02	0.00 ± 0.00
	750	2.24 ± 0.31	0.00 ± 0.00	0.00 ± 0.00	0.01 ± 0.00	0.14 ± 0.03	0.00 ± 0.00
Chal	450	1.11 ± 0.25	0.20 ± 0.03	0.00 ± 0.00	0.00 ± 0.00	0.10 ± 0.02	0.00 ± 0.00
	550	1.75 ± 0.12	0.36 ± 0.04	0.00 ± 0.00	0.01 ± 0.00	0.14 ± 0.01	0.00 ± 0.00
	750	2.53 ± 0.10	0.01 ± 0.01	0.00 ± 0.00	0.01 ± 0.00	0.15 ± 0.01	0.00 ± 0.00
Aer	450	1.26 ± 0.16	0.27 ± 0.02	0.00 ± 0.00	0.01 ± 0.00	0.12 ± 0.01	0.00 ± 0.00
	550	1.50 ± 0.15	0.39 ± 0.07	0.00 ± 0.00	0.01 ± 0.00	0.13 ± 0.02	0.00 ± 0.00
	750	2.36 ± 0.17	0.01 ± 0.01	0.00 ± 0.00	0.01 ± 0.00	0.14 ± 0.01	0.00 ± 0.00

**Table 6:** Toxicometric indicators of unfilled and filled vulcanizates determined at the temperatures of 450, 550 and 750 °C.

Filler	T (°C)	W_LC50_ (g/m^3^)						W_LC50M_	W_LC50SM_
		CO_2_	CO	NO_2_	SO_2_	HCl	HCN		
-	450	105 ± 9	14 ± 1	0 ± 0	92 ± 15	7 ± 2	321 ± 44	4.2 ± 0.8	4.15
	550	79 ± 9	10 ± 2	0 ± 0	66 ± 8	5 ± 0	0 ± 0	3.0 ± 0.3	
	750	69 ± 6	645 ± 618	0 ± 0	83 ± 16	6 ± 0	0 ± 0	5.2 ± 0.3	
Sil	450	155 ± 23	14 ± 1	0 ± 0	123 ± 29	8 ± 2	343 ± 61	4.7 ± 0.8	4.89
	550	111 ± 11	10 ± 0	0 ± 0	95 ± 16	7 ± 1	0 ± 0	3.8 ± 0.3	
	750	89 ± 13	1087 ± 640	0 ± 0	115 ± 17	7 ± 1	0 ± 0	6.2 ± 1.2	
Chal	450	179 ± 42	19 ± 3	0 ± 0	156 ± 12	10 ± 2	962 ± 108	6.0 ± 0.9	5.16
	550	113 ± 8	11 ± 1	0 ± 0	88 ± 20	7 ± 0	0 ± 0	3.8 ± 0.3	
	750	78 ± 3	1919 ± 2044	0 ± 0	118 ± 12	6 ± 0	0 ± 0	5.6 ± 0.4	
Aer	450	156 ± 19	14 ± 1	0 ± 0	124 ± 17	8 ± 1	540 ± 73	4.8 ± 0.2	4.96
	550	132 ± 14	10 ± 2	0 ± 0	97 ± 25	8 ± 1	0 ± 0	4.0 ± 0.7	
	750	84 ± 6	1195 ± 1296	0 ± 0	117 ± 19	7 ± 0	0 ± 0	6.1 ± 0.3	

W_LC50_—the maximum toxic concentration of gaseous product, W_LC50M_—the resultant of the W_LC50_ index of individual products, W_LC50SM_—the arithmetic average of W_LC50M_ indices.

## Data Availability

Data sharing is not applicable.
